# Book Reviews

**Published:** 1816-09

**Authors:** 


### CRITICAL ANALYSIS
### *OF RECENT PUBLICATIONS*
IN THE
### DIFFERENT BRANCHES OF PHYSIC, SURGERY, AND MEDICAL PHILOSOPHY.

*De l'Education Physique de l'Homme;* par ML. FRIEDLANDER, D. M. &c. &c. à Paris, chez Treuttel, et Würtz; 1815.

THIS, if not a very profound, is a very pleasing and interesting work. The nature of his subject necessarily involves the author in a great deal of matter that is superficial and common-place; but we do not recollect to have met with a treatise on *hygieine* which has less to answer for, on the score of jejuneness, than the one now before us. We are, moreover, surprised at the idiomatic correctness, and

 even even eloquence, with which Dr. Friedlander writes in the language of a country of which he is not a native. There are to be found some passages, and even pages, in his book, which are, if we may be allowed to judge, master-pieces of French composition.

One of the first topics on which our author treats may serve to excite a smile among many of his readers; for we are not much accustomed, on this side of the water, to *grave* discussions respecting the best possible combination of circumstances for the propagation of the race, or (excepting by a Shandean mystic) to hear the question agitated whether " y'a-t-il à prendre, pendant l'acte qui contribue le plus immédiatement à la propagation, des précautions, afin de favoriser la naissance d'heureuses dispositions." This discussion, however, ends as it should do; and Dr. Friedlander tells us that of all the institutions that the contrivances of man have been able to devise, that of marriage promises the most in favour of progeny, inasmuch as it insures the most watchful care over the physical and moral education of children.

What period of the year is principally prolific? is another discussion of our author's, the conclusions from which are not of sufficient moment to command our notice: in this part of the book, however, we meet with some observations on the subject of monstrosities, and on the gradual diminution both of these and casualities to mothers and new-born infants, which are not without considerable interest.

" The celebrated Professor Chaussier (we are told) took particular notice, during the space of nearly five years, of the cases of deformity which happened in the *Hospice de la Maternité*, where accidents, if any where, are likely to occur; and the result of his observation was, that out of 23,293 infants, there were only 132 which were ushered into the world with any marks of external deformity: thirty-seven of these had some defect or lameness of the feet, which is the most common of all defects, and occasioned, with most others, without doubt, (as he conceives,) by the practice of mothers, who are admitted into this institution, of frequently tightening their dress for the purpose of concealing their pregnancy. Among thirty-four children (the Professor goes on to remark) who were deformed, as to the shape of the head or the vertebral column, there was not to be found a single instance of resemblance, in any degree whatever, to frogs, apes, rams with horns, devils *(diables)*, or other such-like beings, about which so much noise was made in former times, when fancy supplied the place of observation. Indeed the very extent of the deformities was a mutilation or displacement of natural parts, and this, in the majority of instances, palpably referable to the causes just named."

We have a pleasing note of Dr. Friedlander's appended to the the above statement of Chaussier, pointing out the gradual, and, indeed, subsequently to the year 1789, rapid and striking, diminution of casualities to lying-in women, and infants at birth, in the Lying-in Hospital at London, a circumstance which he attributes to "an improved regulation in the concerns of the house, and other accessory *(accessoires)* causes."

In the second chapter, Dr. Friedlander supposes that much benefit would accrue to the community at large, in reference to the physical management of children, were a census to be taken by each individual accoucheur of the length, breadth, height, and weight, of every child which he was instrumental in bringing into the world.

"These observations (he says), repeated in different countries, in respective families, and among the several classes of the community, would indicate the improvement or deterioration of the race, and by what particular circumstances such variations might be accompanied."

For our own parts, we think that there is somewhat of inapplicable precision in suggestions and plans of this kind, and that we should be likely, by following such courses, to lose sight of generals in the search after particulars,—a fault which is, in our own minds, not an unusual attribute of foreign physiology. We shall, however, present the reader with the following table of proportionate weights of new-born infants, made, upon a large scale, at the institution to which allusion has just been made.

"Out of 7077 infants, born between the middle of the year x* and the last of July 1806, which were weighed with the greatest care,

| | | | | |
|---|---|---|---|---|
| 54 | were found to weigh from | 1 | to 1½ | pound. |
| 69 | ........................ | 2 | .. 2 | idem. |
| 164 | ........................ | 3 | .. 3 | idem. |
| 369 | ........................ | 4 | .. 4 | idem. |
| 1317 | ........................ | 5 | .. 5 | idem. |
| 2799 | ........................ | 6 | .. 6 | idem. |
| 1750 | ........................ | 7 | .. 7 | idem. |
| 463 | ........................ | 8 | .. 8 | idem. |
| 82 | ........................ | 9 | .. 9 | idem. |
| 5 | ........................ | 10 | .. 10 | idem." |

But we must not, according to Dr. Friedlander, rest satisfied with the measurement and weight of children newly born: it is necessary to follow up their progression and in-

* Meaning, we presume, of the Republic, which, dated from A. D. 1792, makes the period alluded to commence in 1802, and comprehends the term of four years.—Edit.

crease crease in a regular series; and, by such means, we should judge, from comparative estimates, whether nature were performing her functions in due course, or whether there were some impediments or interruptions to healthy accretion.

"M. Schwarz (he tells us) measured and weighed one of his own children, after the following manner:

| | inch. | lines. | pounds. |
|---|---|---|---|
| At first he was found to measure | 18 | 8..to weigh | 6 |
| the end of 8 days | 20 | 2 | 7½ |
| 3 weeks | 20 | 8 | 8½ |
| 4 | 20 | 11 | 8¾ |
| 5 | 21 | 3 | 9¼ |
| 7 | 21 | 8 | 9¾ |
| 9 | 22 | 0 | 11 |
| 11 | 23 | 3 | 11¼ |
| 13 | 23 | 7 | 11⅜ |
| 5 months | 24 | 0 | 13½ |
| 6 | 27 | 7 | 14 |

The chapter in which these calculations and observations are met with, is terminated by the following division of the period of infancy into three epochs.

"The first, which commences at birth, and terminates about the sixth month, seems to be principally destined for suction and simple respiration. In this epoch is especially to be noticed a predominance of fluids over the solid part of the system, of lymphatics over blood-vessels, and of cellular substance above muscular fibre. The head is large, the heart bears a proportion to the body of eight to one over the adult state, the pulse beats from 120 to 140 in a minute, the senses and the understanding seem to be in a state of inactivity, and sleep is the principal want of life.

"The second epoch, more complicated and active (*plus orageuse*), commences with the process of teething, at about the seventh month. At this period begins the necessity for a new kind and mode of nourishment; and the developement of corporeal powers invites likewise to something more of energetic exercise. This is the epoch of the principal derangement of functions; and feverish irritations are now very common attendants on the teething process, by which the several maladies incident to the state of childhood are rendered more severe in their nature, and malignant in their aspect.

"From the time of dentition to the period of puberty, we do not meet with any particularly-marked interruption in the general progress of development. It may be necessary, however, just to notice the second teething, which takes place about the seventh year, and which, although not, for the most part, attended with much irritation of the frame, has, nevertheless, some influence over over its physical character. Between the first and second dentition, the efforts of the system are mainly directed towards the completion of the general functions; the digestion gradually accustoms itself to variety of aliment, the chest becomes expanded, the organs of smell and several parts of the countenance develop themselves, the cartilages are ossified, and new actions take place throughout the whole body; the arterial pulsations are reduced to 80 or 90 in a minute, and in the same proportion is augmented the power of the muscular fibre; the brain approaches much nearer to the adult size; and the animal spirits become, in some measure, subordinate to the voluntary power. Almost all the diseases incident to our earlier years make their appearance and exit during this interval, such as the tinea-capitis, eruptive affections behind the ears, worms, rickets, measles, and small-pox. It is not until after the seventh year, and when the second set of teeth have made their appearance, that the digestive powers show themselves equal to a more substantial nutriment, that the lungs take on the adult colour, and that the male voice becomes a little more characteristic; the sutures of the cranial bones now form, the chin increases in size, the physiognomy acquires somewhat of character, and the parent, or instructor, begins to have some influence over the senses and the intellectual faculties." P. 45–8.

The third chapter need not detain us long. It principally consists of directions respecting the best substitutions of food for the mothers' milk, when that cannot be obtained; and of general rules in reference to nursing. We here meet with a compliment to English mothers, as it relates to the management of their infants. French nursing has in it, he says, too much of tendency to a premature excitation of vivacity, while the German system, on the contrary, proceeds too much upon the plan of quieting and unduly curbing the animal spirits. Dr. Friedlander is an enemy to abrupt weaning. He thinks that the process should commence even from the third month, and, in the general way, not be completed till between the seventh and ninth, about the period of dentition. He is rather unfriendly to the plan of cutting upon the teeth, in order to assist their development, and lessen the irritation occasioned by difficult dentition. "For the most part (he says) it is best not to interfere with the operations of nature." This doctrine, however, carried to an extreme, would go to the prevention of any artificial assistance in maladies in general, and would be fatal to the pretensions of the medical art. In reference to the question of incision, in cases of difficult dentition, we would say that it ought not to be done too early, or for every trivial irritation; but that, when the incision *is* made, it ought to be much more freely and deeper than is generally practised. The objection may be valid to an early and frequent frequent cutting of the gum, that it is apt to leave cicatrices, and thus increase the evil it is intended to lessen.

In the fifth division of his work, the author enters into a more minute detail respecting the chemical composition, and nutritive qualities, of the different aliments that are used, either directly or indirectly, for the sustenance of infants. We meet again here with a little more nicety in the way of calculation and statement of elementary proportions, than we are accustomed to find in this country in practical works; but, deducting something from the merit of the treatise before us, on the score of its being rather too much tinctured with Gallic or German particularities, we find in this, as in many other parts, much that is applicable and instructive. Our limits will not allow us more in the way of extract than an abridgment of the statement which the author introduces at the end of this chapter, of the progress of man, from his original condition of savage simplicity, to that of civilization and complicated wants.

"In the state of nature, the mother suckles her infant for a considerable time. She afterwards nourishes it with the vegetable and animal products of the land, rudely prepared for aliment. Man, in this condition, is not pampered by variety; his digestion is active, his exercises violent. Agriculture soon follows the more simple state of nature. The peasant, however, retains a simplicity in his diet, an uniformity in his habits of life. He has scarcely any call but for the exertion of his animal strength, and his existence is but a small remove from the savage. But families shortly increase;—the village becomes a town, the town is converted into a city. Individuals leave their native places in search of fortune and adventures: upon their return, they introduce among their old associates new tastes, new habits, and new aliments. A desire for change engenders commerce—commerce stimulates to manufactures. Professions commence from this new order of things; and now the intellectual faculties are called into exercise, and assert their superiority over mere bodily strength. In this new and complicated condition of existence, different parts of the human frame are, as it were, put into requisition at the expense of the rest. One calling requires the exertion of the lungs, another more particularly of the head, while, in some again, the muscles are almost solely employed. Under these circumstances infants are born, and while they bring into the world with them the irregularities, defects, and, in some respects, the approach to greater perfection, of their parents, they are, of course, subjected to the same mode of sustenance, and the same general habits."

The evils and remedies of social and civilized life are pursued in this manner, through a considerable extent of detail. Our author all along is full of very pleasing anticipations respecting the eventual mastery of intellect in this contest, as it it were, between the external ills to which man is exposed, and the resources for their counteraction with which he finds himself furnished. He is not, indeed, a disciple of that doctrine which teaches the absolute perfectibility of man in his moral and physical nature, but he thinks that what has already been effected by science, warrants a very favourable inference in respect of what yet remains to be accomplished. On the subject of contagions, we meet with the following observations and statements.

"Besides the constant and inevitable influence of seasons, we are exposed likewise to some other causes of distempers which remain to be noticed, namely, *particular contagions*, which affect the system either through the medium of the lungs or the skin; these are either derived from a-far, and brought to us by the winds, are conveyed from a distance by bodies who have been infected with them from contact, or we receive them by actual and immediate communication with infected individuals. These contagions often produce the necessity of complete isolation. Venice, for example, during the time that she was in the possession of the Levant trade, established quarantine laws in order to prevent the introduction of the plague by means of imported cotton. In the seventeenth century, Paris was visited with this malady, and it became necessary to convey the infected, without exception, into houses at a distance from the city, and to forbid their communication with others; and, when Europe some years since was menaced with the yellow fever, plans of prevention were likewise put in practice. In regard to the contagion of *small-pox*, quarantine laws were never thought of. This poison at one time occasioned the death of one in seven of the whole population of London; at a later period, the proportions came to be about one in ten or thirteen. After the practice of inoculation, at the commencement of the last century, was introduced into Europe from Turkey, and when the most favourable time and circumstances were chosen for the operation, the deaths were not more than one in four or five hundred, of those who were infected in this way. Notwithstanding which, the general mortality from the distemper was not diminished, as there were no laws in force for isolating the subjects of inoculation. England lost at least 21,000 every year, out of a population of a little more than 9,000,000; and it was calculated that the annual deaths in Europe, from small-pox alone, was 400,000. After the introduction of *vaccination*, the police of Copenhagen obliged all its subjects to have recourse to it, and to separate entirely those children from any communication with others, who by any accident had taken the small-pox; during the space of one whole year, there was but one instance subsequent to this regulation of death from small-pox. This measure has accordingly been adopted since in several of the Germanic states, and among others in Prussia, a country in which the medical police always meets with great facilities in its operations. But the circumstances of the recent wars have often interrupted the execution of these plans, so salutary to the general population.

"Although England gave birth to the discovery of vaccination, the practice did not become general in London until the year 1803. According to a census of deaths from small-pox, from the year 1788 to 1797 inclusive; the numbers were found 18,538. From 1803 to the end of 1812 the deaths were only 11,532, which is a diminution of 7006. In 1813, only 898 died, which is a fourth less than the preceding year. A committee exists in that country for the distribution of the vaccine virus, and the number of charged glasses (flacons) distributed last year amounted to 25,394.

"In the last report from the central committee of Paris, an account was communicated of the comparative mortality of those different departments in which vaccination had met with most encouragement. At Strasbourg, the deaths in 1803, were 518 from small-pox; in 1812, only one. In forty-three communes of the department de l'Oise, 13,770 individuals died during the ten years preceding the introduction of the vaccine fluid into France, and in the subsequent ten years only 10,510, which is a diminution of 3260. For this happy result, we are indebted to the noble and active exertions of M. le Duc de la Rochefoucauld. Three fourths of all the children that were born in 1813, have been vaccinated, and it has been ascertained that 3,065,000 individuals have been subjected to vaccination since 1804.

"It is remarkable, that in Asia and among the half civilized colonies of Africa and America, the people have been more eager for, or at least less fearful of the new inoculation than the inhabitants of Europe. The government of the Cape of Good Hope, and that of Ceylon, have already effected the total extinction of the small-pox."

Such are the statements of Dr. Friedlander respecting morbid poisons, and his inference from the whole is, that "it ought to be admitted as a part of the physical management of children, that all those who are affected with any disease which is capable of being communicated to another, should immediately and without reference to circumstances, be separated from other individuals; and, that vaccination, the unequivocally good effects of which are every day visible, ought never to be neglected."

The next chapter is on the subject of exercise; under this division, the author is not less interesting than instructive. He regards every minute peculiarity of a nation and people as more influential upon, and more intimately connected with, their moral, physical, and political, condition than is generally suspected. In support of this position we meet with some remarks and facts, which we are sorry our limits will not permit us to transcribe. Yet, this again gives scope to that theorizing and system-making enthusiasm, which, though common on the continent, has never been admitted by the more sober philosophers of Great Britain. Every evil is to

 disappear disappear before the march of intellect, and every thing is eventually to be compassed by the penetration and powers of man.

The parts of the work which treat on the several senses are not of course susceptible of any satisfactory analysis; suffice it to say, that the reader of the book will find, especially on the subject of the organ of speech and the general theory of language, a good deal that is pleasing and philosophical, if not very new or profound. Here again we find Dr. F. full of sanguine anticipations; he admits, indeed, that the various projects which have hitherto had place for the purpose of improving and simplifying language upon the principles of sound, have not done much, "but the impulse has been given, and it is probable that in no very distant day, some superior understanding shall find out the means for doing away the obstacles which at present impede the art of pronouncing, when simple rules may be developed of universal application. Indeed, if we understand him aright, he goes so far as to suppose the time when philosophy shall have annihilated national and local impediments and disabilities to the expression of certain sounds, such as the continental *th*, and the English intermediate something between *oo* and *u*.

On the subject of stammering, which has recently occupied the attention of several persons in this country, as well as on the continent, we do not find much of novelty, or of matter capable of any practical application. "Amman (he tells us,) makes stammering individuals practise the pronunciation of the explosive (explosives) syllables, such as *pac, pec, tac, toc, tic,* &c." The fact is, that the theory of stammering is inexplicable upon any principles with which we are at present acquainted. When, indeed, we discover any organic malconformations, such as occasion impediments to the labial and dental sounds, we find no difficulty in giving their explication, and pointing out their partial remedies; but, when an actual incapacity of pronunciation exists, so that the individual shall only be capable of a kind of spasmodic or convulsive exercise of the organs of speech, without any visible organic defect of structure, it is extremely difficult to attach any decided opinion concerning the cause of such defect, and we are forced to have recourse to the undefined expression of *nervous* inability. We have, however, recently witnessed a remarkable and almost instantaneous cure of this malady. The individual case to which we allude is not singular, many others have been treated precisely in the same manner, and with the same effect. The process by which the cure is accomplished, is at present a secret, but we we trust it will not long remain so; and when it shall be generally known and acted upon, one example at least will be afforded us in favour of Dr. Friedlander's hopeful expectations.

Such are the outlines of the medical part of Dr. Friedlander's book. In the moral education we cannot be expected to follow him. His two concluding chapters on this head prove him, however, to be a man of sound judgment, united with good taste and correct sentiment; and we do not hesitate recommending to our readers the whole of the performance.

---

### *Medico-Chirurgical Transactions, published by the Medical and Chirurgical Society of London.* Vol. VI.

(Concluded from p. 161.)

*A new Method of Tying the Arteries in Aneurism, Amputation, and other Surgical Operations; with incidental Remarks on some collateral Points.* By Wm. Lawrence, Esq. F.R.S. &c.

In these days, and in a work intended principally for discoveries leading to improvements in practice, we were not prepared for a dissertation of fifty pages on so hackmed a subject as ligatures on arteries. Nor could we withhold our surprise, when we found the attention of the Medico-Chirurgical Society directed to Ambrose Paré, Poiteau, Petit, Morand, and other French academicians. Still less were we satisfied at seeing the errors of the venerable Pott wantonly brought forward, although we might feel some relief by reading the compliments paid to Allison, Hunter, and Jones, yet even these seemed to us equally unnecessary. However, as the mention of the last-named gentleman introduces Mr. Lawrence's improvements, we shall transcribe that passage, glad, at the same time, of every opportunity to do justice to the illustrious dead.

"Dr. Jones, (says Mr. Lawrence,) undertook an experimental investigation of this subject, and has explained it in a clear and satisfactory manner, which makes us deplore his premature death as a loss to science. He has banished the use of thick and broad threads, of tapes, of reserve ligaments, of cylinders of cork and wood, linen compresses, and all the contrivances which, employed as securities against bleeding, only served to multiply the chances of its occurrence. The use of the ligature, therefore, under the modifications suggested by our present knowledge of the processes of nature, may be considered to have arrived at perfection.

"Ligatures, however, being foreign bodies in contact with the surface surface of the wound, must irritate, must cause inflammation and suppuration. In amputations, where it is necessary to secure many vessels, a large portion of the wound is exposed to this irritation; its union is retarded, and considerable pain and spasm are sometimes produced. Are these evils inseparable from the use of ligatures? or is there any plan by which we can avoid them? I think that there is; and I shall proceed to state to the Society the trials I have made, and the experience which my practice has furnished on this subject. A longer delay would have enabled me to ascertain some points more satisfactorily, and to procure opportunities of trying the method in cases to which I have not yet applied it. But several friends have urged me to make the proposal known; and I accede to their suggestion the more readily, as it may lead others to make trials, and thus enable us sooner to appreciate the value of the proposal.

"The method I have adopted consists in tying the vessels with fine silk ligatures, and cutting off the ends as close to the knot as is consistent with its security. Thus, the foreign matter is reduced to the insignificant quantity which forms the noose actually surrounding the vessel, and the knot by which that noose is fastened. Of the silk which I commonly employ, a portion sufficient to tye a large artery, when the ends are thus cut off, weighs between $\frac{1}{50}$ and $\frac{1}{60}$ of a grain; a similar portion of the thickest kind I have tried weighs $\frac{1}{20}$ of a grain, and of the slenderest $\frac{1}{100}$. These ligatures do not interfere with the process of adhesion; and we shall hardly entertain any serious apprehension that substances so minute will excite subsequent irritation and disturbance."

We know not what inconveniences may have occurred at St. Bartholomew's by the common thin ligature of strong twine, but, as far as our own experience goes, we have found none, for we cannot call the remaining of the thread for a few days a matter of any consequence. Often have we seen the whole of the integuments instantly unite where enough has been preserved; and the ligature with ease removed by a suppuration extending no further than its surface. Indeed, to do justice to Mr. Lawrence, some of the cases he relates in the course of his paper were treated in this manner, and, on a comparison, we find little difference in the success of the thread or the silk.

It is not, therefore, worth the inquiry whether this mode of tying arteries is Mr. Lawrence's invention or not. Nor will we question the propriety of making the experiment; but we cannot help taking this opportunity of remarking, that the present volume of this respectable Society is rendered ponderous by too many long papers. The present we include in the number. We feel obliged to add, that with such auxiliaries, we are not surprised at the promise of an annual publication. It is, however, justice to add, that Mr. Lawrence's incidental remarks on some collateral points are many of them deserving of notice.

*Further Observations on the Ulceration of the Cartilages of the Joints.* By B. C. Brodie, Esq. F.R.S. &c.

This is a continuation of a former paper, which we noticed in reviewing the preceding volume of the Transactions of this Society. The author begins by observing, that ulcerations of these cartilages are sometimes the effect of long-continued disease in the joints; and, sometimes, that the disease originates in the cartilages themselves. That, though all ages are afflicted with it, yet that of sixty-eight subjects which he saw, fifty-six were under thirty years of age; the youngest an infant of twelve months, and the eldest a female aged sixty years. Some other remarks follow, on the causes, symptoms, and sympathies attending such complaints, particularly in the hip. The remedies follow, which consist principally of seatons, caustics, or issues, local bleeding, &c. These are no novelties; but, in the inflammatory stage, we very much approve of frequent topical bleedings, particularly with the cupping glasses.

"Perhaps (says Mr. Brodie) the history of diseases can in no way be so well rendered intelligible as by the relation of particular cases. Those of which I propose to give an account in the remaining part of this paper, will serve, at any rate, to illustrate some of the observations which I have now made, as well as the remarks contained in the last communication which I had the honour of presenting to this Society, on the subject of the diseases of the synovial membranes. Whoever will take the pains to compare these cases with each other, and to look for corresponding cases in practice, will, if I am not exceedingly mistaken, be convinced that the distinction of the different diseases of the joints is not a mere matter of curiosity, which may be interesting to the morbid anatomist; but that these diseases are different in their progress, that they produce different symptoms, by which they may be known from each other in the living person, and which indicate the employment of different remedies in different cases."

On this subject, we cannot keep from making a few remarks, which we are the more unwilling to do, as Mr. Brodie speaks of sixty-eight cases, which, we are ready to confess, are more than have occurred to us at a much more advanced period of life, and with opportunities quite equal to the author's. But our principal objection is to the manner in which a young practitioner may be studying in which part of the joint the mischief is seated, or decidedly give an opinion on too slender grounds, whilst his whole attention

 should should be paid to symptoms as they demonstrate themselves. If he finds marks of inflammation from pain, general intumescence, and tensity, especially with an account of previous high health, his whole business should be to reduce the inflammation, at all events by general, and, if necessary, by topical bleeding; by the latter only will he gain much advantage, when the disease is completely formed.

In its more advanced stage, he can only act according to circumstances, and must vary his remedies as the symptoms change; but, in whatever part of the joint the disease may have originated, or may exist at the time of examination, is fortunately of little importance, as the most of the treatment will not be regulated by it, even could we acquire such knowledge.

Three cases follow of inflammation of the synovial membrane, which are introduced by the following remark:

"They show (says Mr. B.) the principal circumstances which occur in this disorder, and the varieties to which it is subject. The first affords an instance of inflammation of the synovial membrane in its simplest form, occasioning little else than an effusion of fluid into the articular cavity. In the second, coagulable lymph was effused, in considerable quantity, into the joint; but active treatment having been employed in the beginning of the disease, before the effused lymph had become organized, it was absorbed, and the knee regained its natural size, and its natural degree of mobility.

"The third case is an example of the effects of long-continued and neglected inflammation of the synovial membrane. The joint was permanently swollen, and stiff, in consequence of the synovial membrane having become thickened, and probably from its surfaces being encrusted by organised coagulable lymph; and, as always happens under these circumstances, the patient was liable to a recurrence of the disease from slight causes. This case also shews how long the synovial membrane may continue inflamed, and how frequently the inflammation may recur without affecting the bones or cartilages of the articulation."

On this passage we shall only refer our reader to our last remark.

Three cases follow in which the cynovial membrane had undergone a morbid alteration of structure. Two of these were cases of the true chronic white swelling, arising from no cause that could be traced, and for which no remedy but amputation could be found. The third appeared the effect of inflammation, which had occurred at different periods: ulceration had taken place, and a small sinus opened superficially. This limb was also amputated.—Cases follow of "ulceration of the articular cartilages." The first of these was in in the hip, and the patient under a complaint in the bowels, probably a symptom of general hectic. We transcribe the following, and shall venture to offer our opinions.

"Mary Anderson, 28 years of age, was admitted into St. George's Hospital on the 6th of April, 1815.

"At this time she complained of intense pain in the right knee, which was particularly severe at night, so as exceedingly to interrupt her rest. The pain was referred principally to the head of the tibia. There was a slight swelling of the joint, having the form of the articulating ends of the bones, and not giving to the hand the smallest sense of fluctuation. The leg admitted of being moved on the thigh, but all motion aggravated the pain.

"No more particular account of the previous history of the case could be procured than the following; that she had laboured under pains of the right knee for nearly six years, which had been occasionally relieved; and that, in the first instance, the pain had been unattended by swelling.

"Immediately on her admission, an issue was made, with caustic, on each side of the patella. On the 9th of April, the pain had very much abated. The issues were kept open by the occasional application of caustic; and the pains very soon left her, and the swelling diminished.

"About the 8th of June, she began to experience a return of the pains in the knee, and, in the course of four or five days, they were so severe as to keep her awake at night. There were convulsive startings of the limb, and the joint was swollen in a greater degree than formerly. The pains increased in violence; and her health began to suffer considerably. On the 3d of July the limb was amputated.

"On examining the knee, some lymph and serum were found effused into the cellular membrane external to it.

"The cavity of the joint contained about half an ounce of thin purulent fluid. The cartilage covering the patella was, in some parts, in a natural state; in others, it had the fibrous structure; and, in others, it was completely destroyed by ulceration, so as to expose the surface of the bone. The cartilage covering the articulating extremity of the femur presented the same variety of appearances. On the inside, there was a spot of some extent, which, instead of cartilage, was covered by an organised substance, resembling the substance of adhesions, but somewhat more dense in its structure, as if the cartilage had been formerly destroyed at this part, and coagulable lymph had been effused on the ulcerated surface of bone, which had afterwards become organized.

"The cartilages of the tibia were ulcerated for a very small extent.

"The synovial membrane, in general, was in a natural state. In some places, it was slightly inflamed. On the outside of the joint, it was inflamed in a greater degree than elsewhere, and thickened, and had begun to ulcerate, evidently in consequence of the the abscess in the joint having begun to make its way to the external surface.

"The bones possessed their natural texture and hardness."

We confess ourselves at a loss to know why no mention is made of bleeding, either topical or general, when the pains returned with such severity as to "keep her awake all night." Whether it is that we are grown timid with age, or bold from experience, we should certainly not have urged amputation, without the failure of the above attempts, before the 3d of July, near a month from the beginning of the returning pains. After that time, no wonder if the patient suffered considerably. By the account given of the examination after amputation, we can see very little that might not have arisen during the last inflammation.

A few successful cases follow, in some of which bleeding, local or general, was used. In all, issues were made, or caustics or blisters applied, and kept open.

*Case of Hernia Ventriculi, from external Violence, wherein the Diaphragm was lacerated without Fracture of the Ribs.* By THOMAS WHEELWRIGHT, Esq. Surgeon.

The subject was a German sailor, in the service of this country. In a state of ebriety, he fell from the top of a stage-coach; and, complaining of great sickness, was conveyed to St. Thomas's Hospital, but, his ribs not being broken, he was discharged by the house-surgeon, and came under Mr. Wheelwright's care.

"At ten o'clock, (says this gentleman,) the following morning, I was desired to see him, and found him under great suffering. He complained of most severe pain in his left side, great difficulty of breathing, violent and continued vomiting, chiefly of blood. Pulse 120, small, tremulous, and irregular; countenance pallid; extremities cold; and temperature of the whole surface of the body below the natural standard.

"By assistance he was raised in the bed, and, upon desiring him to cough, he was unable, as the mere attempt to expand the chest gave him excruciating pain. I satisfied, myself, however, that the ribs were not fractured.

"By the violent efforts of retching, the bandage had slipt off his arm in the course of the night, and a considerable quantity of blood had flowed from the orifice, about the room and bed-clothes; in addition to which, he had vomited from two to three pints of bloody fluid. From these circumstances, I entertained little doubt that some internal viscus had given way from the fall, and that he was sinking from internal bleeding. I merely directed some aperient medicine, which his stomach rejected immediately; and a liniment for his side. He expired the same evening, about eleven o'clock; o'clock; and the following morning I obtained permission to examine the body.

"*Dissection.*—On removing the parietes of the abdomen, the viscera were observed to be but little altered from their natural position, and, contrary to my expectation, no blood was extravasated into its cavity. The pyloric extremity of the stomach was confined in an unusual way, and its coats somewhat inflamed. The other viscera were in a healthy state. On turning back the sternum, a considerable quantity of blood was found in the left cavity of the chest, which amounted, by measure, to three pints and two ounces. By introducing the hand, a substance appeared attached to the diaphragm, which gave the feeling of a torn portion of lung; but, upon a more minute inspection, it was found to be a considerable part of the large curvature of the stomach, protruded through a fissure of the diaphragm, and filled with a sort of half coagulated blood. The lung of the same side was much smaller than natural, and occupied the upper and posterior part of the chest, and was strongly adherent in its whole surface; as was also the case with the right lung. These adhesions were evidently of long standing. Their substance did not appear to have suffered in any proportion with their membranous covering. The heart was of its natural size, and appeared perfectly healthy.

"I was still unable to account from what vessel, or set of vessels, so large a quantity of blood had issued; but, upon removal of the whole from the body, a small semicircular aperture was observed at the lower part of the thoracic or strangulated portion of the stomach, through which the blood had slowly escaped into the cavity of the chest; and thus the very gradual increase of difficulty in breathing was accounted for, which, a few hours previous to his death, was so exceedingly distressing. I may further observe, that the fissure in the diaphragm was in the direction from below upwards about an inch in extent, and inclining towards the left side. The stricture was so complete, as with difficulty to allow the points of the little finger to pass. The lower or abdominal portion of the stomach was perfectly empty, as were the intestines generally."

This is certainly a very interesting case, and such as we believe is not on record. The author very justly expresses his surprise that the person experienced so little sense of pain for the first twenty-four hours: this may, we conceive, be partly imputed to his ebriety; but even this would hardly account for such insensibility, if the inflammation had actually commenced before that time.

*Sketch of the Medical History of the British Armies in the Peninsula of Spain and Portugal, during the late Campaigns.* By Sir James Maccrigor, M.D. F.R.S. Ed. &c. late Inspector of Hospitals, and Director of the Army Medical Board.

No one can doubt the importance of such a paper. We

 have, have, however, given a detail of the army practice in our remarks on several papers which have lately appeared in our Journal. The remainder of Sir James's work may be considered as a very valuable table of reference, one important part of which will be found in our Collectanea. We cannot, however, pass over the following passage, for reasons which, by this time, our readers will well understand.

"The military practitioner, in repeatedly abstracting blood, is not to be guided by quantity, or even by the appearance of the blood, but by the relief procured. If this relief be not afforded by a large bleeding, in a few hours the vein must be again opened; and this is to be repeated again and again until respiration is freely performed, and pain of the thorax removed. It cannot be too strongly impressed on the young military practitioner, that neither blistering, sudorifics, purging, or other remedy, can supply the place of bleeding; nor must he judge by what he has seen in private practice, where this sudden abstraction of a large quantity of blood would, perhaps, be improper.

"Pneumonia prevails more among soldiers than in civil life; and well defined as this disease is by nosologists, it requires the experienced military practitioner to detect it. It frequently happens that the patient, so far from exhibiting the well-known diagnostics, appears to labour under every symptom of oppression and debility. Until strictly questioned, he complains of nothing so little as his breast. The true nature of the disease is not detected without the most experienced and scrupulous examination; nor does it show itself in its natural colours, till the functions of the oppressed and congested lungs are in some degree restored by abstraction of blood. Without this relief, it cannot show itself; for re-action, under such circumstances, cannot take place, and the practitioner is led into the fatal error of treating the disease as *low fever*."

*Statements of the Comparative Health of the British Navy, from the Year* 1779 *to the Year* 1814, *with Proposals for its farther Improvement.* By SIR GILBERT BLANE, Bart. F.R.S. Physician to the Prince Regent.

The skill and benevolence of Sir Gilbert Blane are too well known to require any encomium from us; and we cannot help applauding the perseverance of one who can no longer want fame, in thus bringing before the public such a mass of useful information. We could very much wish it had been contained in a pamphlet by itself, and hope to see it as an appendix to the valuable "Treatise on the Diseases of Seamen." The compilation in which it now appears may be too heavy an expense for those to whom it will be principally useful; and, much as we admire every part, it must appear, to those who are not better informed, as if the

 Society

Society were in want of matter, when treatises of such length make so considerable a part of the present volume.

*Case in which a very large Calculus was removed from the Urethra of a Female without Operation; with Examples of analogous Cases.* By J. YELLOLY, M.D. F.R.S. &c.

*Case of the successful Treatment of the Incontinence of Urine consequent to Sloughing or Ulceration of the Bladder from Injury during Labour, with Observations.* By S. BARNES, Esq. &c.

This paper abounds with useful observations, illustrated by well-related cases. The treatment consists in the application of a sponge contained in an elastic bottle, and applied up the vagina. By these means, the excessive dribbling is prevented; and, every two hours, the catheter is applied. Such are only the outlines of the cure. Every surgeon having such a case under his care, will gladly consult the volume.

*Case of Mortification of the Uterus occurring a few Hours after Delivery, with some Remarks on the Causes that produced it.* By THOMAS GRAHAM, Esq.

This case is principally useful in suggesting the importance of examining every dead body, where the *causa mortis* is at all obscure.

*On the Use of the Lactuca Virosa in Hooping Cough.* By T. GUMPRECHT, M.D. of Hamburgh.

*Further Observations on the Ligature of Arteries.* By BENJAMIN TRAVERS, Esq. F.R.S. &c.

This paper commences with the following paragraph, which we transcribe as a specimen of a pompous mode of delivering a suggestion concerning a fact established more than ten years since, by a most ingenious physician, now, unfortunately, no longer able to maintain his rights.

"If (says Mr. Travers) the arrestation of the column of blood for a time sufficient to admit of its becoming a solid, constituted the security to be relied upon; or if, as formerly supposed, the effect of the ligature were confined to the simple coaptation of the sides of the vessel for a time sufficient to induce their adhesion, the propriety of interfering with it might be questionable. But the facts being established, beyond controversy, that the round ligature divides the inner tunics, and that the wound so produced is healed by a process of adhesive inflammation, it seemed probable that the serviceable operation of the ligature was limited to a period far short of that, in which its liberation from the vessel by an ulcerative process could be accomplished."

We need hardly refer our readers to Dr. Jones's most valuable experiments, nor to our own long remarks on the same,* from which it would seem, as we then ventured to conjecture, that the probable cause of the obliteration of the artery, after the division of the inner coat, is not merely inflammation, the effects of which on arteries are very different,† but a condition of the artery, very similar to what Dr. Jones discovered, when its whole thickness was divided in a certain portion of its diameter. If mere inflammation, and the consequent effusion of lymph, were sufficient, we should find such obliteration in all cases of violently-inflamed arteries. Mr. Travers's experiments confirm this; though his object is to show that Professor Assalini's compressor is inferior to the ligature applied in the manner proposed by Dr. Jones. Had the Professor been acquainted with the result of Dr. Jones's experiments, he would at once have seen that, for the purpose of obliteration, the ligature must be preferred. Mr. Travers's experiments prove, as we might expect, that the compressor, to produce an obliteration, must be kept on till, by preventing the circulation in the vasa vasorum, a considerable injury is produced in the artery, sometimes amounting to sphacelus of that part which is contained within the forceps. When this is accomplished, the artery is brought into the same state of *necessity*, to use the language of Mr. Hunter, as when it is divided, and the same process is set up, that is, obliteration, as the only means of preserving life.

If we were to admit, that the effusion is the mere effect of inflammation, we must be aware that the circulation in the vessel is not prevented by this effusion, as is evident from every part of the experiment: 1st, because the condition, and the degree of adhesion, are insufficient of themselves to stop the progress of the circulation at its customary force; and, secondly, because an increased force, which would be the effect of high inflammation, if it existed, is sufficient to overcome the slight obstruction of the lymph, as was proved by some of Mr. Travers's experiments. The stagnation of the blood, therefore, could arise from no other cause than a provision made in the economy to preserve the life of the animal, under all the various conditions of injured arteries; and the small degree of inflammation discovered in the examination is not greater than is met with in every new action, be the final intent what it may. As we know no better ge-

* See Lond. Med. and Phys. Journals, vol. xiv. and xv.

† See our remarks on Dr. Hodgson's Treatise on Diseased Arteries, &c.

neral neral term for these provisions than the *stimulus of necessity*, we shall adopt it from Mr. Hunter: at the same time, we give Dr. Jones the full credit of tracing the effect of this particular stimulus; and Mr. Travers has confirmed it by showing that, as the compressor is slower in producing lesion in the artery, so the obliteration is proportionally slow; but that, if the lesion is induced, the subsequent effects are not less certain.

*Some Experiments on the Chemical Nature of Chyle, with a few Observations upon Chyme.* By ALEX. MARCET, M.D. F.R.S. &c.

Few branches of chemistry have been so sedulously cultivated in modern times as that which has for its principal object the examination of animal matter; and, as this must, we hope, lead to a more intimate and correct acquaintance with physiological science and therapeutics, we cannot too earnestly applaud this meritorious perseverance, especially among many of our own countrymen, in all such researches.

Dr. Marcet has here chosen for his subject one of the most intricate of all others, the very commencement of assimilation and growth, whence, we must suppose, all the principles, as well as compound materials, of animal matter are derived, whether the creature be of the carnivorous or herbivorous tribe.

We are fully aware of the numerous difficulties which must ever accompany all such experiments as serve to investigate the most intricate and "wonderful chemical powers of the digestive organs;" the "almost instantaneous conversion of food into albumen, and soon afterwards into fibrine, fatty matter, and red particles, with the constant appearance of certain salts, all these substances bearing *certain proportions* to each other." For these reasons, therefore, and "in the present imperfect state of the inquiry," as Dr. Marcet very properly considers it, we shall make but few observations upon these experiments and their results.

When it is considered what a vast quantity of phosphate of lime enters into the animal frame, we are rather surprised to find so little notice taken of this ingredient. No efforts seem to have been made to discover either of the components of this *salt*, as it is termed by chemists, excepting the small portion of lime left in the residua of chyme, after destructive distillation. This operation was not carried so far in the analysis of chyle, which was only heated in a glass tube, while the chyle was "thoroughly burnt in a platina crucible."

We are not apprised of the quantities of chyle which

Dr.

Dr. Marcet submitted to experiment, nor whether the nine parts of *saline matter*, obtained from 1000 of chyle, amounted to grains, or fractional parts of a grain only, and therefore too minute for further examination; for, if this saline matter really admitted of additional inquiry, we must regret that proper means were omitted to obtain more satisfactory results.

It is remarked by Dr. Marcet, that chyle derived from animal food is more prone to become putrid; and even that from vegetable food it will enter into the same fermentative process. Now, as phosphuretted hydrogen is invariably evolved on such occasions, we would suggest this to the consideration of the experimentalist, as the most practical source whence he may prove the existence of either phosphorus or, more probably, its acid, in this important fluid.

The nature of chyle may possibly vary at times in the same animal, even when no change has been made in the food. We draw this inference from a supposition which will be readily admitted, namely, that the process of ossification is unequal; it is more rapid with some animals than with others, and the deposition of the earthy phosphate very perceptibly accumulates in some young animals, while, probably, in the aged, this secretion has either ceased entirely, or the phosphate is carried off from the system by means peculiar to each species.

The carbonate of ammonia, heavy fixed oil, and some other things, evolved, are evidently not educts from chyle, but products arising from decomposition. The source from whence the iron is derived cannot be so easily ascertained in any of the cases described; nor that of the alkaline muriate, found in the chyme of the turkey which had fed upon vegetable food only.

Upon the whole, we hope to see these researches continued, and perfectly agree with Dr. Marcet in trusting, "that our more enlarged views of chemistry, and our improved methods of cultivating that science, will soon throw new light upon this important part of physiology."

At the end of the volume is a note, by the same author, *on the Use of Nitrate of Silver, for the Detection of Arsenic*, &c.

The supposed ambiguity of the operation of this valuable test is here very effectually obviated by Dr. Marcet. We believe, however, that some of the modes of operating, described by the author of the test, Mr. Hume, would supersede the necessity of further examinations. If we recollect justly, justly, that gentleman has recommended *lime** to be joined to arsenic on some occasions; and, if the quantity be sufficient, we presume no *soluble* phosphate could interfere in a well-conducted experiment, in which arsenic is to be detected by silver.

Mr. Hume succeeded also with *barytes*, and other earths, to prepare the arsenic for the silver test. Indeed the process, first announced by him in our Journal for 1810, seems adequate to prevent any error: it consists in converting the arsenic into arseniate of potash, by means of *nitrate of potash.* Upon the whole, while the phosphates can be so readily decomposed by lime, we can most safely rely upon the silver test, the ammoniaco-nitrate of silver, as the best of all methods yet devised for detecting this poison.

Such are the contents of this large volume, to which, if ample justice has not been done in our Journal, we can only plead, in our defence, that neither diligence, nor expence in the selection of able hands, have been spared.

---

*On Burns, and the only safe Remedy for curing them speedily and without Pain in every Stage;* by C. H. Dzondi, M.D. Prof. Publ. Ord. of Medicine and Surgery, Director of the Institute for Surgery and the Diseases of the Eyes, Halle, 1816. (Communicated by Dr. Von Embden, at Hamburgh.)

This little work is deserving of the greatest attention. Dr. Dzondi, in this small treatise, proves cold water to be the first, greatest, and most powerful, nay the only remedy, for preventing the most dreadful consequences in cases of burns, if applied timely, and with perseverance. His conviction of its efficacy is partly founded on a multiplicity of experiments made on animals and on himself, and partly results from his most successful treatment of very important cases; he also attempts to prove it *à priori.*

The first physical effect of a heat exceeding more or less 30 or 40° Fahr. on the organization is an expansion of the fluids contained in the [tæda] cellulosa. This condition admits of two principal gradations: in the first or lesser degree the fluids are only so much expanded as to produce an unnatural distension, but not any laceration in the structure of the solids, which though disturbed in their functions are not totally destroyed. The second degree of the physical effect of heat on the organic structure occurs, when the fluids are so much

---

* Philosophical Magazine, 1812.

expanded, expanded, as to produce a laceration of the solids which are mechanically destroyed when the heat volatilizes the fluids into a gaseous form, particularly in proportion as the penetration of heat is more sudden from its rapid increase.

The physical disturbance produced on the organization is very analogous to that occasioned by contusion; for, as in the latter, the solids are distended and lacerated, so are they in the former expanded by the force of the fluids contained in the cellulosa, rarefied by heat, with the exception only that the expansion and laceration produced in this case, is more general, penetrating, and intense; to which is to be added, the total volatilization of the fluids in the form of steam, vapour, or gas, and the carbonization of the solids produced by the higher degrees. The similarity of the pain, the progress of the disorder, and the remedies to be applied, confirm the analogy. By the distension and lacerations of the solids containing the fluids rarefied by the heat, a sensible pain is produced, or, in other words, an irritation in the nervous system, causing an exalted activity of the organic structure, and, of course, an increased influx of blood. This is principally in the capillary vessels in which every inflammation takes its origin, inflammation being nothing else but an exalted activity of this system, caused by an unnatural irritation and producing new and irregular effects.

If, therefore, this violent irritation is not instantly removed, an inflammation of a peculiar character takes place.

The most important peculiarity of this inflammation, besides its long continued sensible pain and the copious secretion of lymph, is doubtless its violent operation upon the whole nervous system, and the reaction caused by it; as no other local irritation seems to produce such a sensible and painful stimulus, nor more obstinately resists every remedy. The violent commotion in the nervous system appearing in the form of a symptomatic fever is only the extension of the local inflammation over the whole nervous system, which particularly suffers so intensely, because by the violent operation of the heat, the nerves of the part burnt are so lacerated in their inmost texture, that the most sensible pain and an unceasing irritation is spread over the whole, not to be perfectly overcome by opium, bleeding, or any other remedy, till the local stimulus is deprived of its irritative power, by the only efficacious remedy in this case, viz. the cold.

Though in burns, as every where else in nature, no exact gradations and definition respecting the different degrees of violence can be given, yet the same may be arranged under four heads.

The

The lower stage takes place when heat operates so slightly upon the surface of the body as to produce a painful irritation, increased influx of the fluids, heat, redness, and an inconsiderable swelling, without any encreased secretion of lymph in the cellulosa. Nature in this case, by means of her exertion to remove every unnatural obstacle, soon restores the equilibrium in healing this inflammation, as soon as the irritation of the nerves and the roused activity of the plastic system subsides, without the assistance of art, or any general reaction of symptomatic fever, except in very few cases, and then but trifling.

In the second stage the irritation of the burn continues long enough to cause an enormously increased activity in the nervous system, and still more of the exhalent vessels of the skin, which shows itself by the secretion and excretion of lymph. A blister filled with an aqueous fluid is raised, which without bursting soon dries up, or, on being opened, heals by exsiccation, and the forming of a new epidermis without suppuration. The inflammation raised is similar to that of the serous membranes. In case the surface of the skin is deprived of its epidermis, it forms analogous to the serous membranes in an inflammatory state, exudations of lymph, which adhere to similar neighbouring organs.

In the third stage the heat has produced its effect, so long and so violently as not only to affect the exhalent vessels, but also the fibrous structure of the skin, causing an inflammation of the same, which in suppuration and cicatrization follows the course of inflammation in the serous membranes, but is aggravated partly by the pain still continuing, and partly by the greater quantity of lymph, which, at first in particular, is still secreted with the pus.

A complete destruction of the organic forms, by a degree of heat, which, by its violence and duration, not only expands the fluids in such a manner as entirely to dissolve, lacerate, and disorganize, the solid parts, establishes the fourth stage; which, if the destroyed structure of the solids, by the partly remaining, but altered, fluids are kept in a state of softness, leads to a gangrene; but, if the too great degree of heat has volatilized them, to a sphacelus.

Different as the three last stages are with regard to local, they are similar with respect to the general, disturbance they produce in the constitution, which is frequently far more dangerous than the local. The general symptoms are those of fever, viz. heat, rigor, restlessness, head-ache, thirst, spasms, altered pulse, convulsions, delirium, and often death. The danger depends on the degree of heat, its duration; the tenderness and sensibility of the organ or of the constitution in general, its habitual or accidental disposition at that period, the situation and position of the affected part, and its sympathies with contiguous or remote organs.

The inflammation caused by burns, has, like all others, if it finishes its full course, three periods.

The first is the phlogistic, the state in which the nervous system in general is unnaturally irritated by the pain from the violent expansion, and put into an inflammatory disposition from the very attempts of the plastic system to preserve or restore the parts. The duration of this period is from a few seconds to an hour in proportion to the irritation, and the reaction roused by it. If assistance is procured at this period, all the consequences of the burning, be it ever so violent or extensive, may be prevented, unless, from the intensity of heat, the organic parts are instantly and perfectly destroyed, or the application of heat is continued. However, this is only to be understood of local inflammation. When the violence of the irritation has caused a general inflammation of the nervous system, it runs on under the form of what is called an inflammatory fever, in three periods of three times seven days, and, if sufficient assistance is not given, often proves fatal in the seven days of the first period.

The second period in this as in every other inflammation, is the plastic or restorative attempt. In this period, the plastic vessels, urged by the violent irritation, secrete an unnatural fluid, which at first consists of a clear serum or lymph, but afterwards, according to the difference of the inflamed organ, may be partly pus, lymph, or phlegm, &c.; but, in deeply penetrating burns, is always pus, in consequence of the fibrous structure of the organ being attacked. In its regular course, the injured surface is not so considerable as to require a longer time for the formation of the epidermis, three times seven or nine days is the period in which cicatrization takes place, and the formation of the dry scurf, if not prevented by art, is performed. The fever rarely attends this second period of inflammation.

The third period, that of the crisis, comprises in this kind of inflammation, the duration of the adhesion of the scarfskin to the injured part till the scar is formed; and in the regular course usually takes up from seven to nine days; which period, however, may be shortened or lengthened by the activity or tardiness in the functions of the organism. In forming our prognosis of the possibility and degree of recovery of the injured part, regard must be had to the following points:—

1. To

1. To the degree of heat.—The lower the same, the more favourable under otherwise similar circumstances.

2. The duration of the operation of the heat upon the organism.—A less degree of heat operating for a longer time will produce a far more disadvantageous disturbance than a higher one operating upon the same part for a shorter time, as by the longer continuance of the irritation the nervous system is shaken in its inmost forms. [The sympathy is more violent and more universal.]

3. The kind and nature of the vehicle of heat.

4. The condition of the local injury.—The more sensible the organ, the greater the danger; the larger the extent of the burn, the more important are the attending circumstances, and the worse dangerous consequences to be apprehended.

5. The general disturbance of the nervous system is, in most cases, of still more importance than the local injury.—The more violent its reaction, such as fever, spasms, &c. the more dangerous the case.

6. Under perfectly similar circumstances, every thing depends upon the period of the inflammation, or the length of time elapsed since the moment of the burn taking place.—Every important disadvantageous consequence may be prevented, and in most cases, at least in all those of the three first degrees, the injury can be perfectly repaired, be the latter ever so violent, provided no considerable destruction of the nobler organs has been occasioned, if assistance is procured in the first minutes after the accident.

7. In the prognostic, regard must be had to the exterior circumstances of the patient.

### *Appropriate Method of Healing Burns.*

The only and proper remedy to the application of which both nature and instinct lead us, is the opposite extreme, viz. cold, which, applied betimes and with perseverance in a sufficiently high degree and fit vehicle, has such a general vigorous effect as speedily to obviate every local and general consequence and effect of heat, complete disorganization only excepted; so that in the course of a few hours it establishes a perfect cure.

The best vehicle for the cold is water, an article to be procured every where, speedily, and in abundance, besides its being, with respect to form and application, the most convenient, easily to be kept in the requisite degree of temperature, as easily modified and renewed, deserving of recommendation; in short, the best, the readiest, and the cheapest.

For the water to produce its full effect, it ought to be applied plied in the very first stage, that is to say, when the reaction roused by the heat, has not as yet produced any unnatural secretion of lymph, and it is only at this time that all and every consequence of burns can be prevented. Even, when by the violence of the heat the cuticle is instantly raised, if cold is directly applied, it will not suppurate, but dry up; and the scab, after remaining about twenty-one days, will fall off; if cold is not applied in the very first stage of inflammation, the extent of that process will be progressive, In the beginning of the second period, the cold will still be of essential service, by removing in a few minutes all pain, and in a few hours every ill consequence, except the blistering, which will disappear as soon as a new epidermis is formed; if the blisters are already burst, and the surface of the skin secreting the lymph lies bare, the cold will not only speedily remove all pain, but put the bare surface in a few hours in a condition that a new epidermis will be produced within twenty-four and forty-eight hours, without any secretion of lymph; even several hours after the accident, and in general, as long as any pain is felt in the injured part, the application of cold is of benefit in stopping the effusion of lymph, and changing the burn into a common wound. If the pain has already fully ceased, the application of cold is superfluous, if not hurtful.

With respect to the duration of the application, the general rule is to continue it as long as any pain is felt in the injured part; of course, it must be different in proportion to the violence of the heat and the disturbance thereby occasioned; we must not, however, be deceived by a freedom from pain for a quarter or even half an hour, or a whole hour, nor excite it again by motion, warmth, or irritating influence; the patient must rather be kept quiet, cool, and avoid every irritation. With respect to the degree of cold to be applied, it is sufficient to take the feeling of the patient for a guide. If the pain gives way to the cold, it is sufficient in degree; as soon as the pain returns, the degree of cold must be increased. A too low degree of cold, for instance, that at or near the freezing point, would be noxious, and on large surfaces, such as the abdomen, produce a bad effect upon the intestines. In most cases, a cold of 12° Ream, seems to be sufficient.

The best and most convenient way of applying cold water is the local or general bath; less suitable are fomentations or pouring the water upon the injured part, these methods being troublesome on account of the necessity of frequent changes, though they are unavoidable in burns of the face and head. However, the fomentations and the pouring of the the water, if used, must be continued without any intermission, so that the pain may not return for a single moment. Burns in the mouth are treated by taking repeatedly cold water into the mouth; those of the throat, by frequent drinking and fomentation. The bath must be kept cold by a frequent change of water. The patient must avoid a pendant posture of the injured limb, be kept quiet, and only drink a cooling beverage.

If this method of treatment is timely adopted, every other remedy may be quite dispensed with, and the open wounded and destroyed places be managed like common wounds. The suppurating and burned spots must not be dressed with any irritating salve or ointment, but only with pure linseed oil, kept warm, and care be taken to prevent adhesion of the parts.

This may be sufficient for the local injury. If the inflammatory affection of the nervous system should require a particular treatment, it can be no other than the antiphlogistic. As a pure suffering of the nervous system is the object, narcotic remedies, and particularly opium in large doses, will be the best antiphlogistic. Should the dose given not produce any effect, and the pain still continue, it must be speedily enlarged, particularly with children. With robust plethoric persons, bleeding and the whole antiphlogistic method may be adopted.

The fever, in the succeeding periods, may be treated quite in the manner of a pure inflammatory one.

The question, In what manner the cold performs the healing of the burn? Mr. D. endeavours to solve in the following manner:—

The cold, if it operates in continuation, (not momentarily, for then it encreases action,) has a double effect on all organic bodies; it deprives them of a part of this caloric, lessening thereby their distension, increases the contraction, and of course brings the parts into closer contact. In this manner it removes the too great warmth of the burnt part, lessens the unnatural distension and pain, increases the connection of the parts, contracts the vessels and the cellulosa, prevents the accumulation of the fluids, and thus operates in opposition to the heat.

The second effect of the cold, partly resulting from the former, consists in a partial or total suppression of the vital activity of the organism, or that part on which it operates in particular. The cold at the same time reduces the sensibility and activity of the nervous system, and thus also alleviates the pain.

The violence of a preternatural irritation of the nervous system system stands in an opposite proportion, with the duration of the same. Every reaction is most violent in the beginning, and, like the vibrations of a touched chord, gradually decreases in force, till it is entirely lost, and the original state returns again. If, therefore, the cold water removes the irritation for a sufficient length of time, the new and increased action of the vessels disappears of itself, and inflammation with it, as there can be no inflammation without an irritative cause.

The recovery of the sound condition, the formation of a new epidermis under the dried scurf, as also the removal of the latter and the formation of the scar, are only to be accounted for by the general organic power of nature, and the general vital principle.

The above article, received from our German correspondent, we lose no time in inserting, though we have not had an opportunity of perusing the work.

The most important part is the unreserved recommendation of cold water perpetually renewed at the common temperature. This we give on the authority by which we receive it, only adding, that we have never found any inconvenience from cold application after these accidents. The directions for the temperature are simple enough, depending entirely on the sensations of the patient. If the water is too cold, it will soon be raised by contact with the injured surface to the wished-for temperature; and when it exceeds it, which will be known by the same test, the water should be agitated, and, if necessary, fresh added.

There are two important omissions in that paper as we have received it: first, the danger of scalding or burning a considerable part of the skin, however slightly, and, however slight the immediate effects may seem, though it may be fairly surmised from paragraph 4, p. 243, is not impressed with sufficient force; next, the cautions against the contraction of the granulations, and the subsequent union of parts which ought to remain separate, as two fingers, or the neck with the chin, is not sufficiently dwelt upon. But we have inserted the paper as soon as we received it, that we might not for a moment withhold the advice given at a period when the most important remedy is probably the only one at hand.—Ed.

---

*Edinburgh Medical and Surgical Journal*, No. XLVII. for July, 1816.

Art. I.—*Observations on the Utility of Blood-letting as the principal Remedy in Continued Fever; with some Cases of an Epidemic, in which it was practised with uniform and remarkable Success*. By John Allan, Surgeon, Royal Navy.

Another proof of the advantage of blood-letting. Though practical knowledge is the most important, yet an attention to causes is often not less necessary, in order to teach us the means of prevention.

"At the time (says Mr. Allan) that the disease which gave occasion for these remarks made its appearance, the ship had been about three weeks in Portsmouth harbour, and we afterwards remained there about five, that is, altogether, from the middle of April to the beginning of June last; during which period a certain number of the men were daily on shore on leave, an indulgence attended, as usual among sailors, with various excesses, especially frequent intoxication. It was, however, in very few instances, that the disease took place immediately after the application of these causes; for, though a few cases did occur early in the month of May, while we remained in the harbour, the greatest number, and some who had the most severe attacks, were not taken ill till several weeks after we had left that situation; and, indeed, we were scarcely without one or more cases of fever on the sick list till after the middle of August. The disease, therefore, must have owed its origin to some other cause besides the temporary irregularities just alluded to; and marsh miasmata, the most frequent cause of uncontagious fevers, appear, in this instance, to have been no less active than usual. Our ship was so situated, during the time I have mentioned, as to be within forty or fifty yards of the extensive slimy mud-banks on the Gosport side of Portsmouth harbour, which are continually uncovered at low water, and from which, when the sun's rays were not interrupted by clouds, one might sometimes perceive exhalations rising in the form of a whitish vapour.

"But, besides the obvious and unavoidable opportunities thus frequently presented for the application of this generally active cause, there was, in the nature and character of the epidemic, something that evinced its origin from such a source. In several cases it manifested a distinct and decided tendency to assume the type of an intermittent, and, although the paroxysms were never regularly completed in all their stages, yet the occasional, and somewhat regular, recurrence of cold fits often summoned my attention, and gave reason to apprehend the formation of a regular remittent or intermittent, as the issue of the cases. Had such symptoms led to a relaxation in the use of the lancet, or had the cure been chiefly confided to bark, or to any other remedy, I cannot help believing that such must, in some cases, have been the result. I do not mean to say that bark was wholly useless, or without good effect; on the contrary, it will appear to have been administered, in some of the following cases, with decided advantage; but the disease was in general so speedily cut short by the bleeding, as to leave the strength of the system very little impaired, and to render quite unnecessary the use of any tonic whatever.

"The lancet was used as freely in this disease as it usually is in the yellow-fever, and sometimes most extensively, on account of other unmitigated symptoms, when the pulse was both frequent and feeble, and when other apparent signs of extreme debility were were no less conspicuous; in short, (I wish it to be particularly remarked), when circumstances, commonly supposed to forbid its use, were certainly not wanting."

We have transcribed this passage, because we conceive the writer of the paper either not sufficiently attentive to the events within his cognizance, or not sufficiently explicit in his relation. In England, marsh miasma is by no means the most common cause of fever, especially in the situation here described. A muddy shore, covered at every returning tide, (excepting between the tropics, and not always there,) has not for a long succession of years been sufficient to excite intermittents; and even those marshes, which, during the vernal and autumnal months, are usually in a state of high vegetation, have been less productive of ague for some time past. But, in the present instance, there is nothing to authorise such a suspicion. Gosport is not known as an aguish coast. None of the patients had more rigor than usually attends continued fever, and the disease affected none of the commissioned officers. From all this we should conceive the cause of the fever was introduced, either by fresh men admitted with cloaths imbued with the effluvia of hospital or poor-house fever, or contracted by some of the men permitted to go on shore, and probably sleeping in some lodging-house contaminated from such a cause. We mention this, because, from the mistaken notion of the author must follow an inattention to that most important part of a medical officer's duty, namely, the preventing the introduction of such a cause of fever. If men are permitted thus to go on shore (and we are aware it is absolutely impossible entirely to prevent it,) there should be licensed houses for all the purposes for which they procure leave, and proper attention should be paid to the healthiness of each; fresh recruits should never be admitted without previous ablution and change of dress: even those who sleep on shore should perform a short quarantine, and their cloaths left on deck or hung to yard-arms during the first night.

Of the cases, we shall only remark, that, though in most the pulse was small, yet this was very properly no objection with the author to free bleeding. One patient was bled during the rigor. We trust the embarrassment thus produced will be a caution with Mr. Allen to defer that operation till the commencement of the heat. On this subject, we recommend him to peruse Dr. Dickson's paper contained in a former number of the Edinburgh Journal, with the authorities in our own, which we have offered on the same.

Art.

### Art II.—*Case of Mortification of the Appendix Vermiformis Cæci, occasioned by a Human Tooth found in its Cavity.* By William Briggs, M.D. Liverpool.

Our remarks on this paper will be best expressed by transcribing the author's postscript.

"P. S. Since the above statement was written, my attention has been directed to a case in the London Medical and Physical Journal for this present month (February 1816), where mortification was occasioned by a *chocolate-nut,* lodged in the entrance of the appendix vermiformis. To this case, as resembling the one just detailed in many important particulars, I would beg leave to refer my readers. If they will be at the trouble to compare the dates, it will probably strike them as singular enough that two such cases should have occurred so nearly at the same time. In the one, no history could be traced as to the length of time that the nut had been swallowed, farther than that the patient "*had experienced a similar affection of his bowels about a year before.*" So also in the other, after the cause had been ascertained by dissection, several of Mr. D.'s friends could recollect that, at times, for a year or more, he had, when walking, been obliged to stop short by a sudden attack of pain in the right iliac region, which, however, soon went off, and no farther notice was taken of it by himself or others. None of his own family ever heard of his having swallowed a tooth. One gentleman, indeed, who had been his intimate from a very early age, *thinks* he recollects something of the kind having been told him by Mr. D. when they were at school together, but he does not speak positively; and my friend, Mr. Perry, the dentist, to whom I showed the tooth, says it *must have belonged to the second set,* for it is one of the upper bicuspides teeth, which do not exist in the infant set. Mr. P. authorizes me to say, 'that he cannot believe it to have been one of Mr. D.'s own teeth, but that it must have been swallowed, as any other moderately-sized substance is liable to be, in his food.' And, to show how very possible this is, he states, 'that, the week immediately before Mr. D.'s death, a well known individual in Liverpool happened to cut down upon a human tooth, in a *hot roll* which he was going to eat at breakfast.' He farther says, that 'he had been in the habit of examining Mr. D.'s teeth, and that they were more free from decay or disease of any kind, than is general perhaps; that he had lost one or two, which he thinks were molares, but that, to the best of his recollection, the bicuspides were all in their places, and sound; that the tooth in question had more the appearance of having belonged to a *female*; that it had a covering of what is called tartar, which occupied the natural cavity betwixt the points of the grinding surface, obliterating them as points; that this extended on one side about two-thirds of the length of the fang, indicating, as he infers, that it was lost by this accumulation, and a gradual retiring of the alveolar process, as is usual in advanced age; but he will not take upon him to say, that the tooth may not have acquired its covering while it was lodged in the intestine or appendix.'"

These conjectures are all fair, and we may add another, that the tooth might have been formed there, as we find them with similar substances, sometimes are in various parts of the body, at which they could not have arrived by swallowing.—See Ruysh, tom. ii. Adversar. Anatom. also Baillie's Morbid Anatomy.

Art. III.—*Fatal Case of Intestinal Disease, with the Appearance on Dissection;* by GEORGE NESSE HILL, Surgeon, Chester.

THIS is one of the most interesting cases we have ever met with, and related in a manner no less creditable to the writer. Of this, we need give no better proof than that it occupies seven closely-printed pages without wearying us in reading, and with little or no unnecessary verbosity. For this reason, we shall not attempt to offer an epitome. We regret, that like too many others of the kind, from the time the history commences, as appears by the examination after death, nothing could have been done, had even the medical attendant been able to explore and operate upon the whole of the diseased parts.

Art. IV.—*Observations on the Cure of Cancer of the Womb by Excision;* by F. B. OSIANDER, Professor of Midwifery in the University of Gottingen.

OUR readers will recollect an appendage to this paper, which we published in one of our late numbers. The subject is so important, that we have reserved it for our Collectanea, and the whole shall appear in our next number, extracted from the German Journal of "Learned Observations," published at Gottingen. As the operation has, we believe, never been attempted in England, we shall feel it our duty to give it every publicity; and, as every medical reader must frequently have witnessed the dreadful complaint for which so bold a remedy is proposed, we shall make no apology for offering a few remarks of our own.

Art. V.—*On a newly-constructed Sound for the Purpose of discovering the Stone in the Bladder, accompanied with a Plate.* By JAMES BARLOW, Surgeon, Blackburne, Lancashire.

SEE our Journal, No. 204, or Vol. XXXV. page 81.

Art. VI.—*Reply to Dr. Monro's Observations on the Discovery of the Action of the Oblique Muscles, attributed to Dr. Mayow.* By G. D. Yeats, M. D. Fellow of the Royal College of Physicians, London.

This paper is written with all the candour and perspicuity which distinguish the author's other performances. That Dr. Monro has no claim to any part of the discovery of the office of the two sets of oblique intercostal muscles can admit of no doubt, because it was marked by several modern anatomists. But Dr. Yeats's laudable zeal is not satisfied without giving to Mayow his mead of praise. This is very laudable and fair in the present instance; yet, we should be careful how we detract from the moderns by producing the labours of the ancients. Dr. Hunter never failed to disgust his class when he endeavoured to prove, that Hervey was not the discoverer of the circulation; and Beddoes, notwithstanding his many eccentricities, never so much disgusted his readers, as by attempting to wrest the discovery of oxygen from Scheel and giving it to Mayow. In this the public judged right. There is nothing in nature which may not have occurred to experimental philosophers; but a hint, nay, a discovery, passes for little till the various results are placed in such order as to teach, as far as we can learn, the whole chain of phenomena dependant on such discovery. Imperfect views of a distant light will glimmer and disappear, their existence will be doubted and gradually forgotten, till a progressive knowledge of the sources from which they are derived, and of the means of approaching them are unravelled; the philosopher who teaches us this is the inventor. Under this view, Hervey and Scheele have the undoubted claim to their discoveries, as much as Davy to potassium, though the probability of such a substance had been hinted by Kerr.

But, in the present instance, the whole and every part was discovered and demonstrated by Mayow; and this, as well as several other controversies which we need not repeat, only show the danger of rendering professorships hereditary.

Art. VII.—*On the Use of the Poultice in Internal Inflammations;* by William English, Surgeon, London.

This modest appeal in favour of an apparently simple remedy is deserving of every consideration. It is certain, that physicians are too apt to overlook topical, and surgeons, general or constitutional remedies. Mr. English gives many practical instances of the efficacy of poultices in relieving internal inflammation unattended with penetrating wounds.

 "About

"About two years ago, I was seized with pleuritis: my cure was confided to the very kind and skilful management of two eminent physicians; the most approved treatment was resorted to, bleeding, both generally and locally, and blistering, were not spared; but, in defiance of all that was done, for six days the pain and misery remained unabated, when, on the seventh day, a poultice was suggested, and applied in the way I have always seen it the most speedily effectual: a large blistering plaster was laid on for two hours, and a warm poultice succeeded, which was repeated about two hours afterwards. This happily soothed me to sleep, the first time I am sensible of having had that comfort for a week. By the continuance of this remedy, aided solely by a little aperient medicine, in a day or two I was enabled to lie on my side, which hitherto was impossible; expectoration became copious and easy, and in a short time I recovered.

"In March last, a particular friend of mine was seized with an acute inflammatory attack; the pain was entirely confined to the left side. He placed our fingers where the appendix of the diaphragm arises from the lumbar vertebræ, as the part where the most excruciating pain was felt. He was in excessive misery; the least movement or cough was torment to him. Dr. Babington attended this patient with me, and of course every thing likely to subdue the complaint was resorted to. One peculiarity in the symptoms attracted our attention, viz. a considerable expectoration of a beautiful yellow bile, unmixed with the glairy phlegm he was perpetually coughing up. He declares all our remedies were ineffectual until the application of the poultice on the fifth day, which gave him great relief. This was kept constantly warm to the part affected for two days, when the pain was so far abated that he requested permission to lay it aside, its weight being oppressive. He recovered very slowly. I have seen cases that put on all the characters of peritonitis and enteritis, where the stomach was so irritable, that medicines could not be retained for an instant, which did well by the application of a very large blistering plaster, succeeded in two hours by poultices kept constantly *hot*, after which, medicines could be administered effectually."

"I consider the warmth and moisture of a poultice to be much superior to fomentations, and it is attended with less inconvenience and trouble. The only objection is its weight, but this, however, is seldom complained of, until the cause for which it is applied be removed. A bladder, half filled with warm water, is a very good substitute for the poultice; but this is even more complained of for its oppressive weight, and I think less efficacious in all respects. The application of a very large blistering plaster, for an hour or two previous to the poultice, certainly facilitates the beneficial effects thereof; but as blisters, when applied large, are apt to give trouble and pain by the strangury they cause, about one-third of the emplast. lyttæ, with two-thirds pix Burgundica, forms a plaster less liable to this objection, and is even more stimulating stimulating to the skin; of course, more efficacious, when applied for this purpose."

Some other remarks follow, very much to the same purpose; and we trust our readers, with us, have made up their minds to try the effect of poulticing, the first time a fair case of internal inflammation presents itself. The paper concludes with a remark, that the poultice is only considered as the best topical application, not to supersede blood-letting and other antiphlogistic remedies.

*(To be concluded in our next.)*

